# Intimacy across species boundaries: Interspecific allopreening between Spot‐necked (*Stachyris strialata*) and Nonggang Babblers (*S. nonggangensis*)

**DOI:** 10.1002/ece3.9195

**Published:** 2022-08-26

**Authors:** Wenyi Zhou, Zhuyang Zhang

**Affiliations:** ^1^ Department of Biology University of Florida Gainesville Florida USA; ^2^ Florida Museum of Natural History University of Florida Gainesville Florida USA; ^3^ AlpineBirding Sichuan AbsoluteWild Travel Co., Ltd Chengdu China

**Keywords:** behavioral ecology, dominant–subordinate relationship, interspecific allopreening, Nonggang Babbler, Spot‐necked Babbler, Timaliidae

## Abstract

Allopreening occurs in many species of birds and is known for providing hygienic and social benefits. While this behavior has been extensively studied among conspecifics, its occurrence among different species remains little known. Outside of captive environments, only a few records of interspecific allopreening exist. In this study, we describe our observations of the interspecific allopreening behavior between Spot‐necked (*Stachyris strialata*) and Nonggang Babblers **(** *S. nonggangensis*) in a non‐captive environment in southern China. To our knowledge, these observations represent the first record of interspecific allopreening in the family Timaliidae. We suggest that this understudied behavior is most likely related to the dominant–subordinate relationship between these two species: either the dominant species preening the subordinate species to assert dominance or the subordinate species preening the dominant species to reduce tensions by appeasement. We also suggest interspecific allopreening may not be as rare as we thought if we study this behavior under circumstances where different species are close to each other. This study contributes to our understanding of not only the potential mechanism(s) behind interspecific allopreening but also the behavioral ecology of the vulnerable Nonggang Babbler.

## Graphical abstract

Interspecific allopreening is a rare and understudied behavior in birds. In this Nature Note, we describe our novel observations of the interspecific allopreening behavior between Spot‐necked (*Stachyris strialata*) and Nonggang Babblers (*S. nonggangensis*) in a non‐captive environment in Guangxi, China. We suggest that this understudied behavior is most likely related to the dominant‐subordinate relationship between these two species. We also suggest interspecific allopreening may not be as rare as we thought if we study this behavior under circumstances where different species are close to each other.

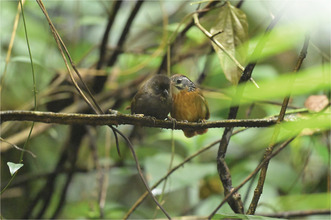

## 摘要

异体理羽(allopreening)能为鸟类提供清洁和社交方面的好处，在多种鸟类中曾被记录。虽然鸟类种内不同个体间的异体理羽行为已经得到了大量研究，但是关于种间异体理羽行为的报道仍然较少。在非圈养环境中，仅有少数种间异体理羽行为的记录。在本研究中，我们描述了在中国南方一个非圈养环境下，斑颈穗鹛(*Stachyris strialata*)和弄岗穗鹛(*S. nonggangensis*)之间的异体理羽过程。据我们所知，本次观察是林鹛科(Timaliidae)中种间异体理羽行为的首次记录。我们认为，这种较少被研究的行为很可能与两个物种间的主从社会关系有关:主导种通过为从属种理羽以维护其主导地位，或从属种通过为主导种理羽以示好主导种从而减少冲突。我们还认为，在不同物种距离较近的环境下，种间异体理羽行为可能不如想象中那么罕见。本研究不仅有助于了解鸟类种间异体理羽的潜在机制，同时有助于了解易危物种弄岗穗鹛的行为生态学。

## 1 INTRODUCTION

Grooming and preening behaviors between different group members occur in many social animals, particularly in mammals and birds (Baker & Aureli, 2000; Carter & Leffer, 2015; Gill, 2012; Lewis et al., 2007). In birds, allopreening refers to the phenomenon when one individual preens the feathers of another (Cullen, 1963). The adaptive significance of this social behavior includes providing hygienic and social benefits. On the one hand, allopreening improves birds' plumage condition by reducing ectoparasites (Brooke, 1985; Villa et al., 2016). On the other hand, allopreening maintains pair bonds between mated pairs (Gill, 2012; Kenny et al., 2017), improves cooperation during parental care (Gillies et al., 2021; Takahashi et al., 2017), and reduces aggression between conspecifics (Lewis et al., 2007; Radford & Du Plessis, 2006). The hygienic and social benefits are not mutually exclusive (Kober & Gaston, 2003; Radford & Du Plessis, 2006).

While intraspecific allopreening occurs in many species (Barbour & DeGange, 1982; Gill, 2012; Harrison, 1965; Radford & Du Plessis, 2006), interspecific allopreening has only been sparsely documented in a few species in non‐captive environments: between Black Vultures (*Coragyps atratus*) and Crested Caracaras (*Caracara plancus*) (Ng & Jasperson, 1984; Palmeira, 2008; Sanabria, 2015; Souto et al., 2009), a Razorbill (*Alca torda*) and multiple Common Murres (*Uria aalge*) (Walsh et al., 2001), a Royal Spoonbill (*Platalea regia*) and an Australian White Ibis (*Threskiornis molucca*) (Mo, 2016), and a Mitred (*Psittacara mitratus*) and Monk Parakeet (*Myiopsitta Monachus*) (Cortés, 2017). This behavior also occurs in several species of Icterids, which actively perform allopreening invitation displays toward other species and receive allopreening from them (Garrett & Molina, 2005; Hunter, 1994; Post & Wiley, 1992; Selander & La Rue Jr, 1961; Verbeek et al., 1981).

Despite these records, we still lack an understanding of the mechanism(s) underlying interspecific allopreening and the associated allopreening invitation display. Here, we report the first documented record of interspecific allopreening between two gregarious babblers in the family Timaliidae in a non‐captive environment. These species are Spot‐necked (*Stachyris strialata*, hereafter “SNB”) and Nonggang Babblers (*Stachyris nonggangensis*, hereafter “NB”). While SNB occurs from southern China to southern Sumatra (Collar & Robson, 2020), NB is only found in the limestone forest in southern China and northern Vietnam (Jiang et al., 2020). In regions where these two species co‐occur, they often form mixed‐species aggregations in bird blinds (W. Zhou, Z. Zhang, *pers. obs*.), which are bird‐feeding stations built by local people as a form of bird photography tourism.

## 2 FIELD OBSERVATIONS

On February 3, 2022 and February 4, 2022, we observed three allopreening bouts between SNB and NB in a bird blind (22°29′57.0′′N 106°57′24.6″E) near Nonggang National Nature Reserve in Guangxi, China (see Table 1 for a summary). Following Gill (2012), we defined an allopreening bout as continuous preening motions involving two birds, with motions separated by 10s considered different bouts. During all three allopreening bouts, allopreening only occurred from SNB to NB, not vice versa. Because no birds were marked, we could not determine if the participants in each allopreening bout were the same individuals. We only observed the third allopreening bout from the beginning to the end. We did not observe how the first and second bouts began. Apart from these three interspecific allopreening bouts, we also observed frequent intraspecific allopreening among NBs.

**TABLE 1:** A summary of the three allopreening bouts

Allopreening bout	Date	Time	Duration (seconds)	Species offering preening	Species receiving preening	Body parts preened (observable only)	Allopreening invitation display	Temperature (°C)
1	February 3, 2022	10:10 a.m.	15	SNB	NB	Nape	No	9.4
2	February 4, 2022	10:41 a.m.	141	SNB	NB	Back, flank, and breast	Yes	12.2
3	February 4, 2022	11:11 a.m.	102	SNB	NB	Nape and head.	Yes	12.2

**FIGURE 1:**
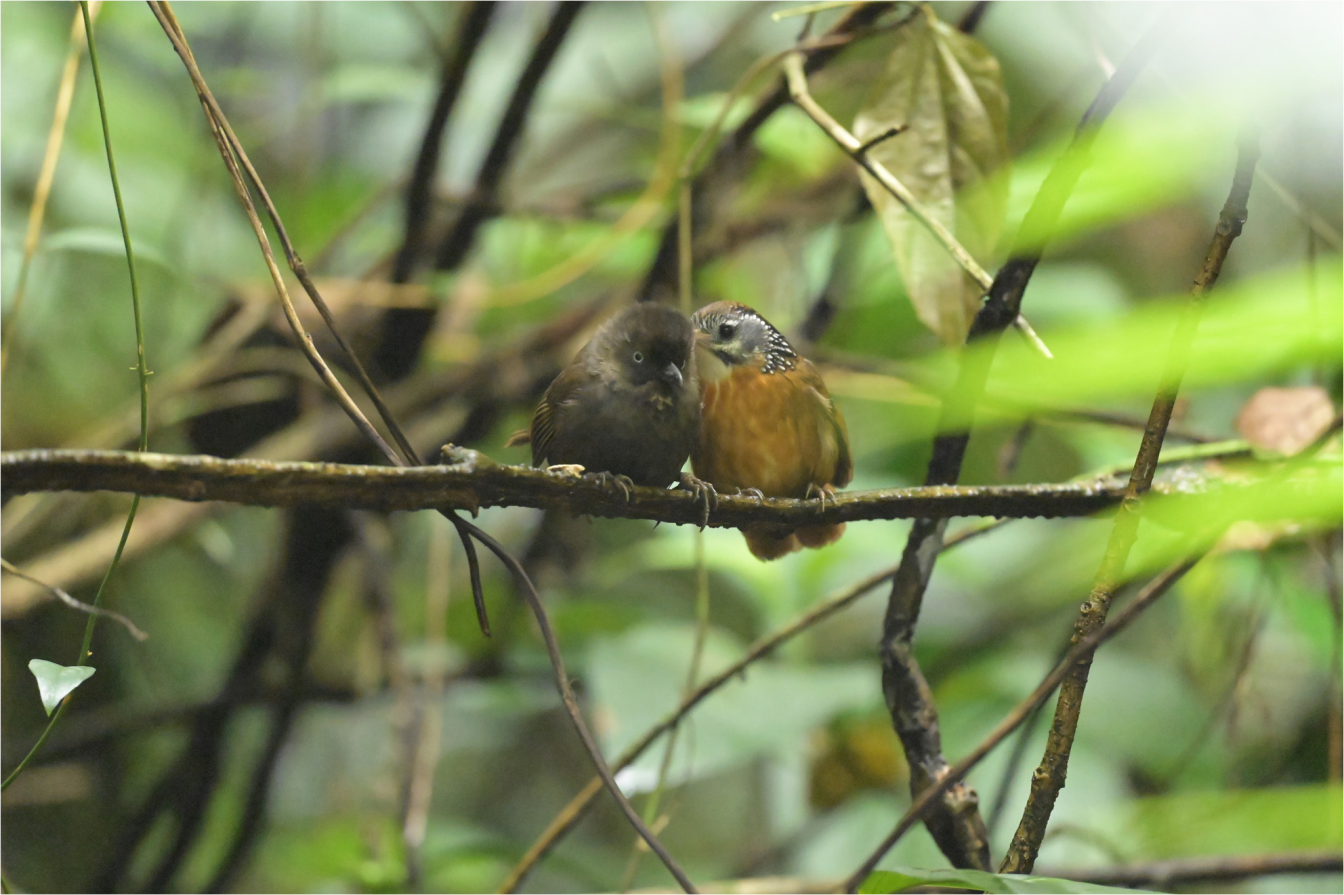
A Spot‐backed Babbler preening the nape region of a Nonggang Babbler.

Our first observation lasted for 15 s at 10:10 a.m. on February 3, 2022. One SNB and one NB perched side by side on a vine branch 0.8 m above ground (Figure 1). During this observation, the SNB preened the nape region of the NB several times. The NB did not react to the preening. Instead, it only turned its head slightly away from the SNB as it was being preened. When the bird blind owner began tossing mealworms to feed the birds, the allopreening stopped and both birds flew off to eat the mealworms. Throughout the observation, a group of self‐preening NBs perched 0.5 m above the allopreening pair without any interaction

**FIGURE 2:**
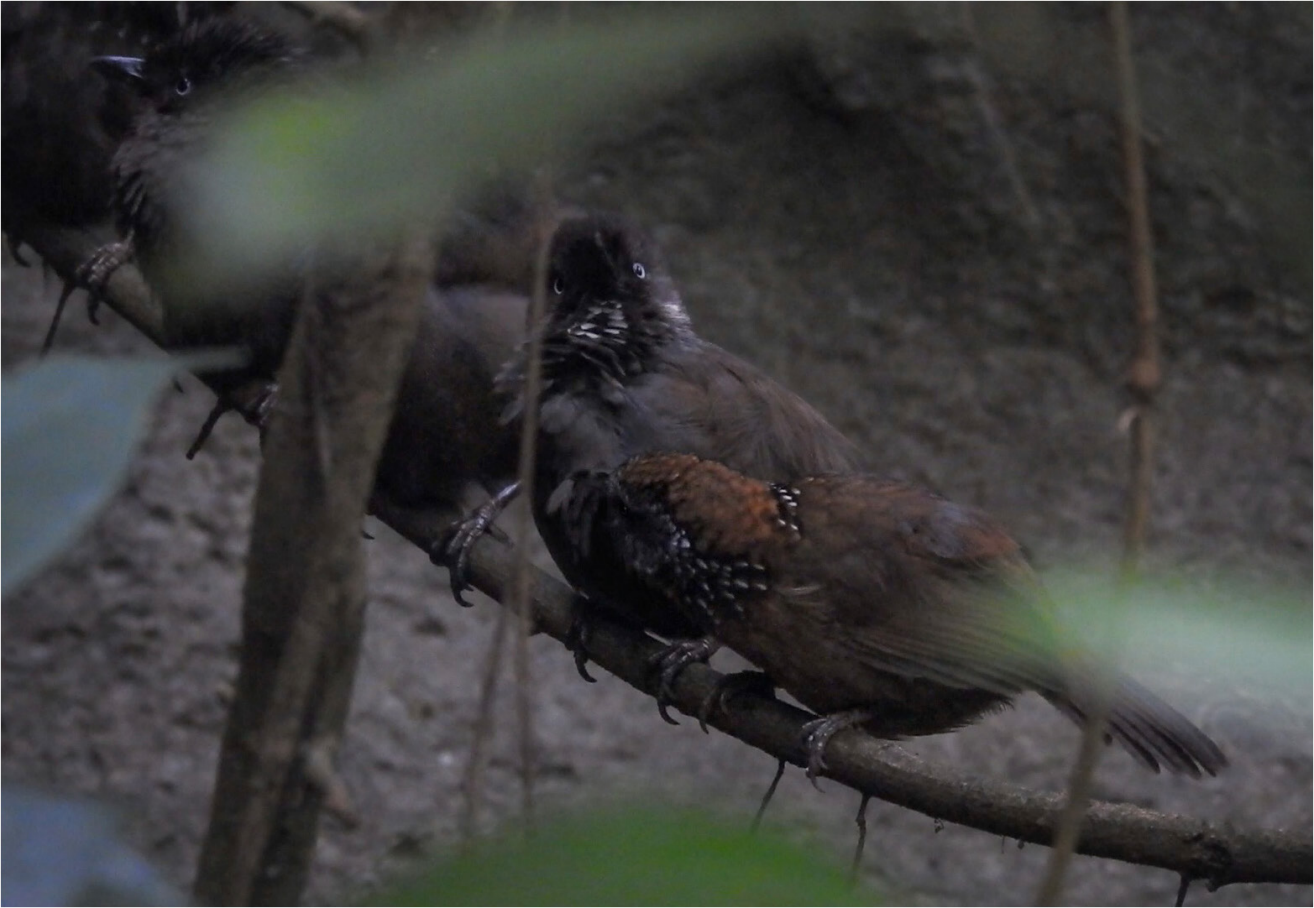
Head‐up allopreening invitation display from a Nonggang Babbler to a Spot‐necked Babbler.

Our second observation lasted for 2 min 21 s at 10:41 a.m. on February 4, 2022. One SNB perched next to a group of 7 NB on a vine branch 0.7 m above ground. During this observation, the SNB only preened the neighboring NB. The SNB initially preened the back and flank regions of the NB while the NB self‐preened. At 1 m 40 s of the observation, the NB stretched out its neck toward the SNB, with its throat and breast regions exposed and feathers of those regions erected (Figure 2). This posture was similar to the head‐up allopreening invitation display described in Scaly‐breasted Munias (*Lonchura punctulata*) (Moynihan & Hall, 1954), Brown‐headed Cowbirds (*Molothrus ater*) (Selander & La Rue Jr, 1961), and Jungle Babblers (*Argya striata*) (Gaston, 1977). We also observed this display in NBs when they allopreened intraspecifically. Upon noticing the invitation display from the NB, the SNB began preening solely on the breast region of the NB for 24 s. When the NB stopped the display and lowered its head, the SNB shifted back to preening the back region of the NB. The allopreening bout ended when the SNB moved slightly away from the NB. Both birds then began self‐preening. They both flew off the vine branch when the other birds at the bird blind suddenly began alarm‐calling. Eventually, the NB flew back to the vine branch and rejoined the other NBs.

The third observation lasted for 1 min 42 s at 11:11 a.m. on February 4, 2022. At the beginning, one SNB approached one resting NB from behind while other SNBs and NBs remained in the vicinity. As soon as the SNB landed next to the NB, the latter stretched its head forward and erected feathers on its head, nape, and throat, possibly performing another allopreening invitation display. The SNB then began preening the NB. This allopreening bout included the nape and head regions of the NB. It also included other body regions that we could not clearly determine. The allopreening bout ended when the SNB pushed its left leg against the body of the NB, which then flew away before being followed by the SNB. During the entire observation, the NB remained on the same perch (0.2 m above ground), with feather erection and eye pinning. We also observed the SNB nibble many times right after preening the NB, as if it were eating the ectoparasites from the NB.

## 3 DISCUSSION

During our observations, the allopreening from SNB to NB included many self‐accessible body parts such as the flank and breast regions. While allopreening on self‐inaccessible regions such as the head and nape tends to serve a hygienic function, allopreening on self‐accessible regions tends to serve a social function (Radford & Du Plessis, 2006). We believe the observed interspecific allopreening instances between SNB and NB is most likely related to the dominant–subordinate relationship between the two species. Such a relationship can lead to allopreening between conspecifics of different social dominance rankings (Miyazawa et al., 2020; Radford & Du Plessis, 2006). Therefore, it may also lead to interspecific allopreening if different interacting species have different social dominance rankings (Ng & Jasperson, 1984). This possible link between interspecific allopreening and dominant–subordinate relationship has been studied in Icterids (Post & Wiley, 1992; Rothstein, 1980; Scott & Grumstrup‐Scott, 1983). However, the mechanism behind interspecific allopreening in these species is likely different because they actively seek preening from other species through chasing followed by allopreening invitation displays (Selander & La Rue Jr, 1961). We did not observe such behavior from NB toward SNB.

In social animals, intraspecific allopreening can occur both from subordinate to dominant individuals and vice versa. On the one hand, subordinate individuals are known to preen dominant ones to reduce social tensions (Kutsukake & Clutton‐Brock, 2006; Radford & Du Plessis, 2006). The allopreening behavior in this case serves a conciliatory function. If SNB were subordinate to NB, the purpose of its allopreening could be reducing potential conflict by pleasing the dominant NB. On the other hand, dominant individuals are known to preen subordinate ones to assert dominance (Harrison, 1965; Miyazawa et al., 2020). The allopreening behavior in this case perhaps originates from an agonistic intention of the dominant preener (individual offering preening) to attack the subordinate preenee (individual receiving preening). However, instead of fleeing from the preener, the preenee performs the allopreening invitation display by exposing its vulnerable area to the preener, perhaps to express submission. The preener then replaces its original intention to attack with allopreening as an outlet behavior (Harrison, 1965). If SNB were the dominant species, the purpose of allopreening could be asserting dominance over NB.

Whether the observed allopreening behavior was the subordinate species pleasing the dominant species or the dominant species asserting dominance over the subordinate species, it might have occurred because of the need to establish a flock hierarchy (Scott & Grumstrup‐Scott, 1983). With both species seeking access to the concentrated food resources at the bird blind, an established dominant–subordinate hierarchy would clarify the pecking order in this mixed‐species aggregation. While we could not determine the exact dominance rankings of these two species, we observed them chasing each other off the feeding platform. Measuring aggressive behaviors such as chasing/supplanting will allow future research to determine the dominant–subordinate relationship between these two species, therefore improving our understanding of the interspecific allopreening behavior between them.

Besides the dominant–subordinate relationship, we also considered the possibility of a cleaning association, in which the preener eats the ectoparasites from the preenee through allopreening, and hybridization, in which two allopreening participants form a mated pair. However, we found insufficient support for both hypotheses: cleaning associations have rarely been recorded between two bird species (Sazima et al., 2012) and hybridization has never been recorded between SNB and NB (McCarthy, 2006). However, because our study does not eliminate the possibility of these hypotheses, future research on interspecific allopreening should consider them as possible alternative hypotheses that need to be tested.

With an increasing number of instances being discovered, interspecific allopreening may not be as rare as we thought. Perhaps, we only perceive it being rare because we have not studied this behavior in circumstances where it most frequently occurs. Future research should study this behavior in locations where birds are near each other, as allopreening is more likely to happen under such circumstances (Cullen & Ashmole, 1963; Harrison, 1965; Morales Picard et al., 2020). These locations include resting and bathing areas, captive environments, roosting sites, and feeding sites such as bird blinds and feeders, carcasses, and clay licks. Also, future research should focus on social species, particularly species that display clumping behavior and ones that form mixed‐species associations and aggregations with others. Lastly, it is essential to be attentive because interspecific allopreening may be easily overlooked without being taken into consideration.

## AUTHOR CONTRIBUTIONS

**Wenyi Zhou:** Conceptualization (equal); investigation (lead); methodology (equal); writing – original draft (equal); writing – review and editing (equal). **Zhuyang Zhang:** Conceptualization (equal); investigation (supporting); methodology (equal); writing – original draft (equal); writing – review and editing (equal).

## CONFLICT OF INTEREST

The authors declare no conflicts of interest.

## Data Availability

This study does not include any data (video footage of the allopreening instances is publicly available at https://doi.org/10.5061/dryad.mw6m90609).
